# Externalized Rectal Duplication in a Newborn - A Differential Diagnosis of Meningomyelocele

**DOI:** 10.7759/cureus.51306

**Published:** 2023-12-29

**Authors:** Bianca M Silva, Ana L Onofre, Giovana T Comes, Renata B Antunes, Bruna N Tedesco

**Affiliations:** 1 General Surgery, Hospital das Clínicas de Botucatu - Universidade Estadual Paulista (UNESP), Botucatu, BRA; 2 Pediatric Surgery, Hospital das Clínicas de Botucatu - Universidade Estadual Paulista (UNESP), Botucatu, BRA

**Keywords:** meningomyelocele, neonatal surgery, congenital anomaly, rectal duplication, bowel duplication, duplication of digestive tract

## Abstract

Rectal duplication is a rare congenital anomaly with many clinical presentations, being mostly asymptomatic. Treatment consists of a surgical approach with a good prognosis. We are reporting a case of a full-term female newborn who presented with a mass externalized through the sacral region. The first diagnostic hypothesis was meningomyelocele, but the neurosurgeon verified peristalsis on examination with a surgical microscope. The pediatric surgery team proceeded with the investigation with barium enema, anal electrostimulation, biopsy, and pelvis MRI, confirming presacral rectal duplication. The patient underwent surgery for posterior sagittal surgical excision, with satisfactory evolution. Cases of rectal duplication are rare and we are unaware of reports of its exteriorization through the sacral region. Such presentation may mimic other diagnoses and should be included in the differential diagnosis of meningomyelocele.

## Introduction

Digestive tract duplication is a rare congenital anomaly, with a reported incidence of one in every 4,500 live births and a slight predominance in male newborns [1]. William Ladd introduced the term "duplication of the digestive tract" in 1937 when he described congenital anomalies with three common findings: a layer of well-developed smooth muscle, an epithelial lining, and an intimate connection to the alimentary tract [2].

Digestive tract duplication can manifest in a variety of forms, which makes a single embryological explanation unlikely. Associated vertebral, medullary, genitourinary, and intestinal malformations suggest a multifactorial process [3]. The exact cause of alimentary tract duplications is not known, but they are believed to occur because of embryological aberrations between four and eight weeks of gestation. Researchers have proposed many theories to explain etiopathogenesis. The split notochord theory is based on the abnormal separation of the growing notochord from the endodermal cells. This theory is mostly cited to explain the occurrence of vertebral anomalies associated with bowel duplication [4].

The patients may be asymptomatic and diagnosed incidentally by physical examination or imaging [5]. The symptoms are nonspecific and vary from minor digestive problems, such as abdominal pain, vomiting, and abdominal mass, which are the most common symptoms and attributable to bowel duplications, to severe outcomes, such as intestinal obstruction, gastrointestinal bleeding, and perforation [6].

Duplications of the small intestine account for nearly half (47%) of all reported duplications of the digestive tract and are most commonly found in the ileum, followed by duplications of the esophagus and colon. Rectal duplications represent 3% of all reported duplications and are most often found in the presacral space, posterior to the rectum [3]. There is a lack of reports of externalized rectal duplication in the literature. We report a case of a newborn (NB) with duplication of the rectum externalized in the sacral region. We discuss the existing data in the literature and draw a parallel with the possible differential diagnosis of meningomyelocele.

## Case presentation

**Figure 1:**
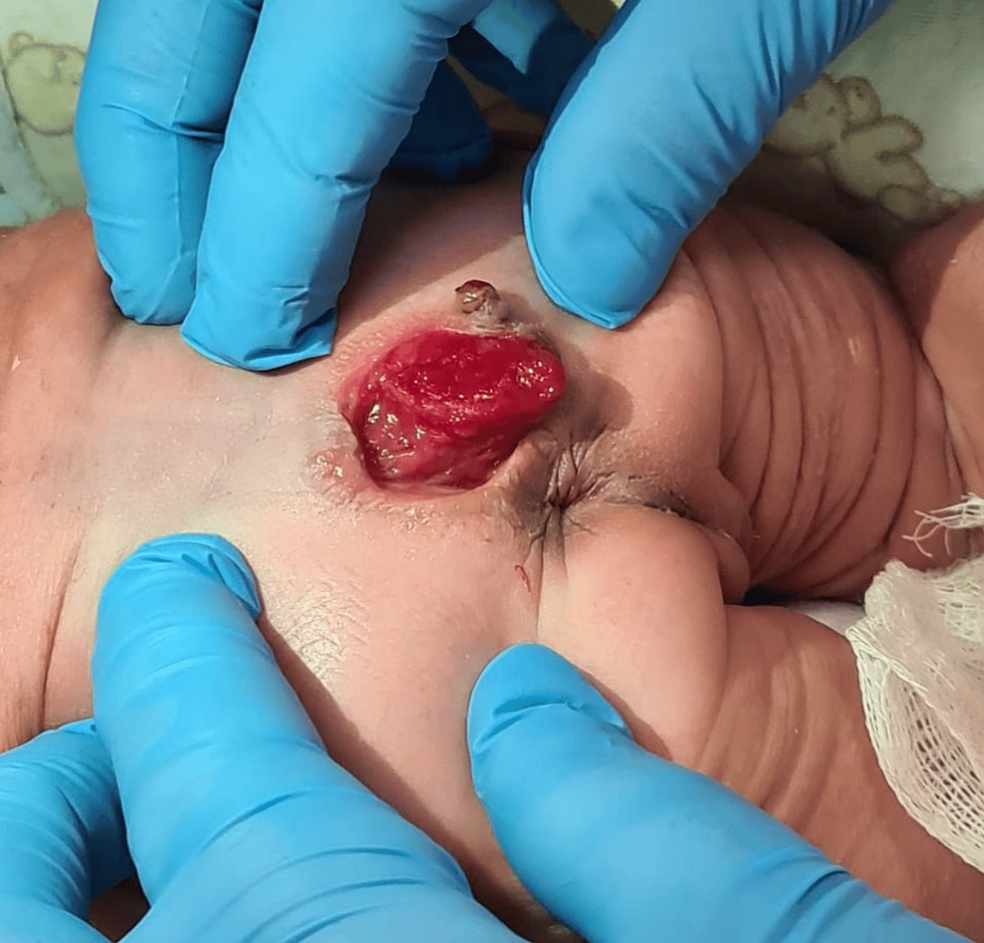
Externalized mass in the sacral region

We present a full-term female newborn, birthed through spontaneous vaginal delivery, presenting with loud crying, good tone, and immediate spontaneous evacuation at birth. The neonatology team observed the presence of a reddish externalized mass in the sacral region with no secretion (Figure 1). On physical examination, the patient was in good general condition, and the anus was topical and pervium.

**Figure 2:**
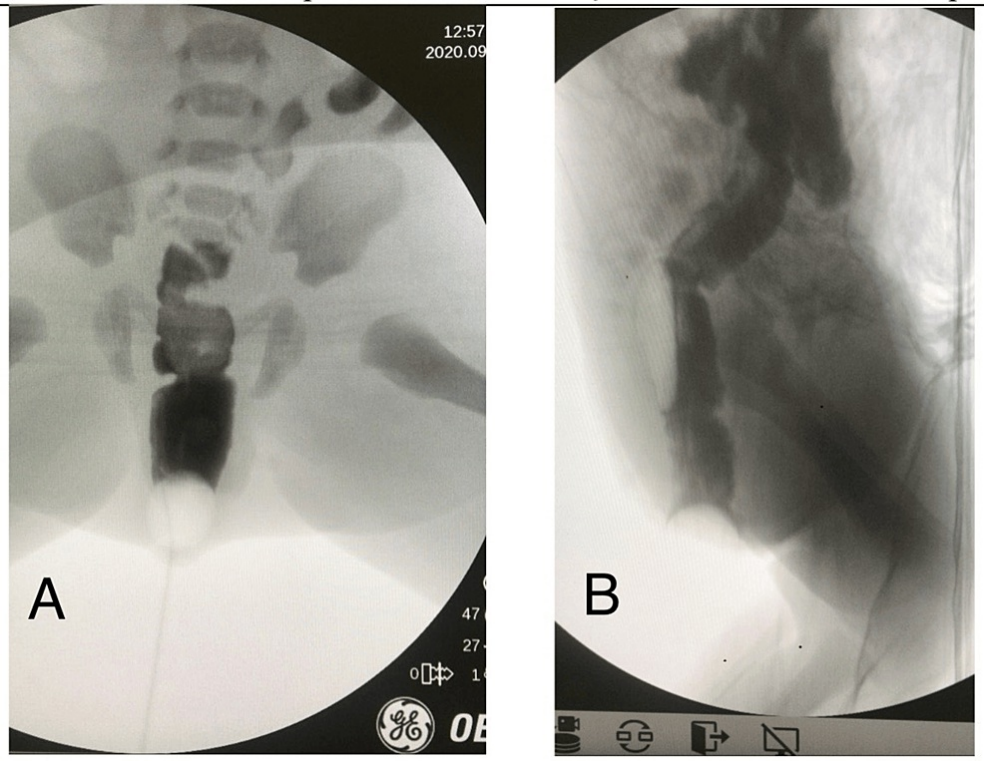
Barium enema showing patency of the anus and no communication with the rectal duplication A: Posteroanterior view; B: lateral view

Faced with the hypothesis of meningomyelocele, the neurosurgery team decided to take the patient to the operating room for more effective evaluation under narcosis, and they verified the presence of peristalsis on examination with a surgical microscope. While still in the operating room, the pediatric surgery team performed a diagnostic investigation with a barium enema (Figure 2), electrostimulation of the anal sphincter, and a biopsy of the lesion.

**Figure 3:**
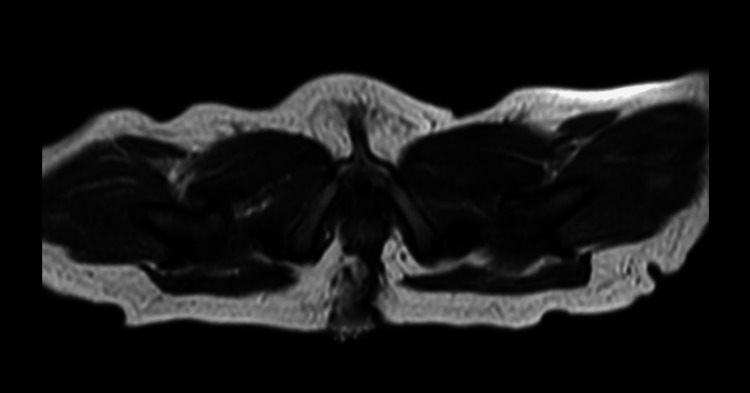
Pelvic MRI showing anomaly presacral with no communication to the rectum on axial view

The biopsy confirmed a histological finding compatible with the large intestine. Subsequent evaluation with pelvic magnetic resonance imaging (MRI) revealed an anomaly presacral in close contact with the rectum but with no fistulous path (Figure 3).

**Figure 4:**
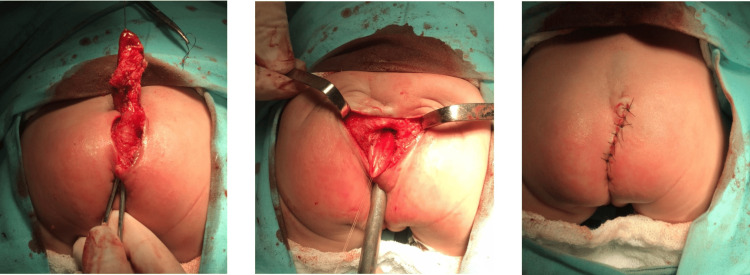
Surgical resection of the sacral primitive intestine

The patient underwent surgical treatment with total resection of the sacral primitive intestine via the posterior sagittal route without intercurrences (Figure 4) and remains under outpatient follow-up with good evolution and preserved fecal continence.

## Discussion

Rectal duplications are rare congenital anomalies, which in 85% of patients are detected prenatally or in the first two years of life but can remain undetected until older age. Among all the duplications of the digestive tract, rectal duplications are the rarest. Considering the studies with the largest number of digestive tract duplications, we highlight Guerin et al. (2012) with 114 cases and Mejaddam et al. (2017) with 107 cases. These studies reported only one case of rectal duplication each [6,7,8].

The number of antenatally detected duplications is increasing, which allows early treatment with fewer complications. Possible complications such as bowel perforation, bleeding, obstruction, and malignant alterations are why all duplications should be surgically treated at the time of diagnosis. The treatment consists of a surgical approach with an excellent prognosis [6]. In cases of rectal duplication, surgery is performed via posterior sagittal, such as in this case, or abdominoperineal [4]. Surgeons have used laparoscopy-assisted resectioning with or without a small laparotomy for uncomplicated cases, but this procedure has not been described in externalized rectal duplication [6,9]. Postoperative complications are rare and nonspecific, such as bleeding, infection, or bowel obstruction [6].

Rectal duplications can mimic other presacral masses, including sacrococcygeal teratoma, meningomyelocele, and dermoid cysts [7]. Even though rectal duplication rarely occurs, it should be considered a differential diagnosis. Most rectal duplications are cystic and located in the presacral space, posterior to the rectum [3], as in this case. However, the rectal duplication reported here is externalized, which is an exceptional finding. There are rare reports of exteriorization through anal prolapse of the mass [10,11], but we have not found any reports of exteriorization through the sacral region.

## Conclusions

Our report adds to the few reports of rectal duplication, which is a rare congenital anomaly with a varied clinical presentation. Early diagnosis and surgical treatment reduce the risk of severe complications and provide an excellent prognosis for patients. With this report, we bring to the literature a new type of presentation, externalized through the sacral region, which should be included among the differential diagnoses, especially meningomyelocele.
